# *catena*-Poly[[[bis­(aceto­nitrile-κ*N*)(4,4′-dimeth­oxy-2,2′-bi­pyridine-κ^2^ *N*,*N*′)copper(II)]-μ-tri­fluoro­methane­sulfonato-κ^2^ *O*:*O*′] tri­fluoro­methane­sulfonate]

**DOI:** 10.1107/S2414314620014078

**Published:** 2020-10-30

**Authors:** Rafael A. Adrian, Diego R. Hernandez, Hadi D. Arman

**Affiliations:** a Department of Chemistry and Biochemistry, University of the Incarnate Word, San Antonio, TX 78209, USA; b Department of Chemistry, The University of Texas at San Antonio, San Antonio, TX 78249, USA; Vienna University of Technology, Austria

**Keywords:** crystal structure, copper(II), coord­in­ating tri­fluoro­methane­sulfonate, tri­fluoro­methane­sulfonate salt, aceto­nitrile, 4,4′-di­meth­oxy-2,2′-bi­pyridine

## Abstract

The central copper(II) atom of the title salt, {[Cu(CF_3_SO_3_)(CH_3_CN)_2_(C_12_H_12_N_2_O_2_)](CF_3_SO_3_)}_*n*_ or [[Cu(CH_3_CN)_2_(diOMe-bpy)(CF_3_SO_3_)](CF_3_SO_3_)]_*n*_ where diOMe-bpy is 4,4′-dimeth­oxy-2,2′-bi­pyridine, C_12_H_12_N_2_O_2_, is sixfold coordin­ated by the N atoms of the chelating bi­pyridine ligand, the N atoms of two aceto­nitrile mol­ecules, and two tri­fluoro­methane­sulfonate O atoms in a tetra­gonally distorted octa­hedral shape. The formation of polymeric chains [Cu(CH_3_CN)_2_(diOMe-bpy)(CF_3_SO_3_)]^+^_*n*_ leaves voids for the non-coordinating tri­fluoro­methane­sulfonate anions that inter­act with the complex through weak hydrogen bonds. The presence of weakly coordinating ligands like aceto­nitrile and tri­fluoro­methane­sulfonate makes the title compound a convenient starting material for the synthesis of novel metal–organic frameworks.

## Abstract 2

The title Cu^II^ complex shows the typical Jahn–Teller distortion of the octa­hedral coordination sphere, defined by the N atoms of a 4,4′-dimeth­oxy-2,2′-bi­pyridine and two aceto­nitrile ligands in the equatorial plane and two O atoms of tri­fluoro­methane­sulfonate anions in the elongated axial positions.

## Graphical abstract

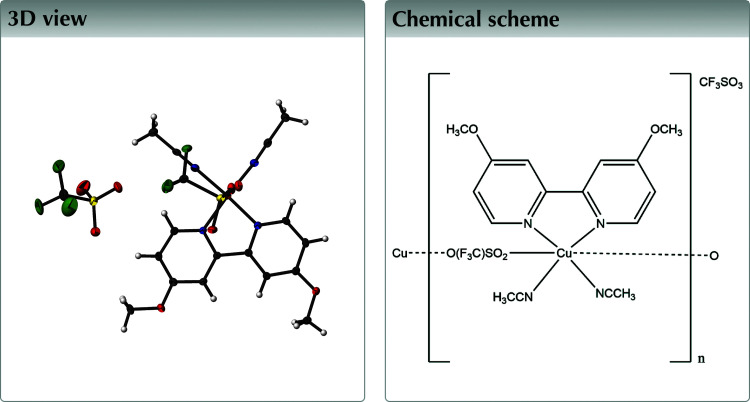

## Structure description

4,4′-Dimeth­oxy-2,2′-bi­pyridines are continuously being investigated for their photoluminescence features (Ravaro *et al.*, 2018 ▸), anti­microbial activity (Drzeżdżon *et al.*, 2019 ▸), good affinity in DNA binding (Anjomshoa *et al.*, 2016 ▸), and anti­tumor activity against human cancer cells (Qin *et al.*, 2019 ▸). As part of our research related to the coordination chemistry of metal ions with bi­pyridine and terpyridine ligands, in the present report we describe the synthesis and crystal structure of the title copper(II) complex salt, [[Cu(CF_3_SO_3_)(C_2_H_3_N)_2_(C_12_H_12_N_2_O_2_)](CF_3_SO_3_)]_*n*_.

**Figure 1:**
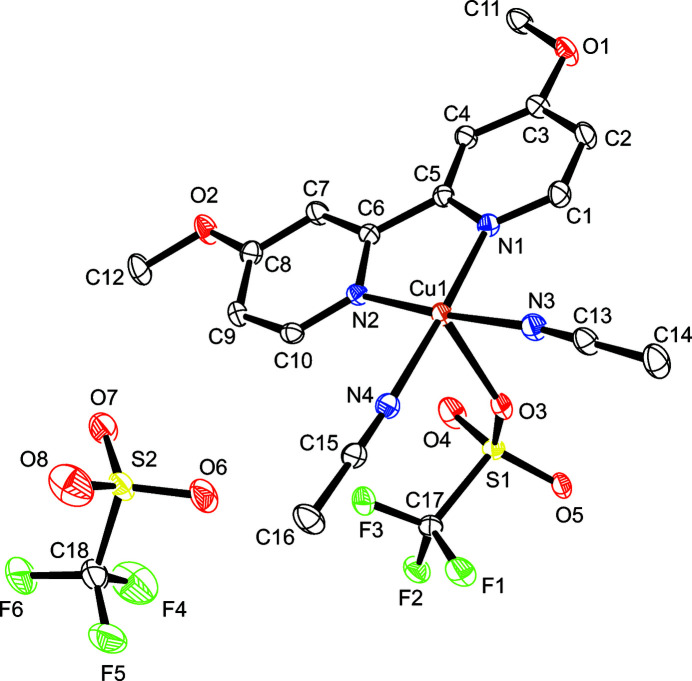
Asymmetric unit of the title compound with displacement ellipsoids drawn at the 50% probability level; H atoms are omitted for clarity.

**Figure 2:**
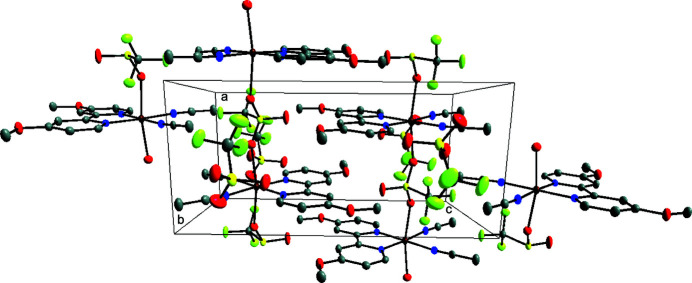
Perspective view of the packed structure of the title complex along the crystallographic *b* axis; H atoms are omitted for clarity.

As depicted in Fig. 1 ▸, the asymmetric unit of the title compound comprises a Cu^II^ atom, one *N*,*N*′-chelating 4,4′-dimeth­oxy-2,2′-bi­pyridine ligand, two aceto­nitrile ligands, and two tri­fluoro­methane­sulfonate anions. The central copper(II) atom exhibits a tetra­gonally distorted octa­hedral coordination environment defined by the N atoms of the chelating 4,4′-dimeth­oxy-2,2′-bi­pyridine ligand and two neutral aceto­nitrile mol­ecules in the equatorial plane and by two O atoms of symmetry-related tri­fluoro­methane­sulfonate anions in axial positions. Although the Cu—N bond lengths with the bi­pyridine ligand are shorter than the Cu—N bond lengths with the coordinating aceto­nitrile mol­ecules, their values are comparable with the reported values of other copper(II) complexes with the same chelating ligand (Fettouhi, 2017 ▸). The aceto­nitrile ligands are bordering on linearity. All relevant bond lengths and angles involving the Cu^II^ atom are presented in Table 1 ▸. The cations in the title complex are aligned into polymeric chains extending parallel to the *a-*axis direction and pack into layers parallel to the *bc* plane, as illustrated in the crystal packing diagram given in Fig. 2 ▸. This arrangement leaves voids in which the second type of tri­fluoro­methane­sulfonate anions are located. These anions are non-coordinating and inter­act through hydrogen bonds.

Graph-set analysis is a method of analyzing hydrogen-bonding patterns in three-dimensional networks. Hydrogen-bonding patterns are classified into one of four pattern designators; rings (*R*), chains (*C*), intra­molecular hydrogen-bonding patterns described as self (*S*), finite hydrogen-bonding patterns described as discrete (*D*). These designators include a superscript with the number of acceptor atoms, subscript with the number of donor atoms, and a number in parentheses indicating the number of atoms in the hydrogen-bonding motif (Grell *et al.*, 1999 ▸).

There are three types of hydrogen-bonding motifs present in the crystal lattice, with numerical values collated in Table 2 ▸. A centrosymmetric hydrogen-bonding ring, 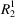 (7), occurs between the O4 atom on the coordinating tri­fluoro­methane­sulfonate anion with two hydrogen atoms on a 4,4′-di­meth­oxy­bipyridine mol­ecule on a neighboring asymmetric unit. The non-coordinating tri­fluoro­methane­sulfonate anion forms a hydrogen-bonding ring, 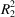 (12), through two C—H⋯O inter­actions with the O6 and O7 atoms. The other oxygen atom, O8, on the non-coordinating tri­fluoro­methane­sulfonate anion, has a discrete hydrogen-bonding inter­action, *D* ^1^_1_(3), with a neighboring coordinating aceto­nitrile mol­ecule.

## Synthesis and crystallization

To synthesize the title compound, 4,4′-dimeth­oxy-2,2′-bi­pyridine (0.105 g, 0.486 mmol) was suspended in 40 ml of aceto­nitrile and stirred for 15 min. Solid CuCl_2_·2H_2_O (0.083 g, 0.49 mmol) was added to the suspension and heated with stirring at 323 K for 1 h. AgOTf (0.250 g, 0.972 mmol) was added to the mixture and stirred without heating for 2 h. After the removal of AgCl by filtration, using a 0.45 µm PTFE syringe filter, the resulting blue solution was used to grow crystals by vapor diffusion with diethyl ether at 278 K.

## Refinement

Crystal data, data collection and structure refinement details are summarized in Table 3 ▸. The highest remaining electron density is located 0.93 Å from atom O3.

## Supplementary Material

Crystal structure: contains datablock(s) I. DOI: 10.1107/S2414314620014078/wm4141sup1.cif

Structure factors: contains datablock(s) I. DOI: 10.1107/S2414314620014078/wm4141Isup2.hkl

Supporting information file. DOI: 10.1107/S2414314620014078/wm4141Isup3.mol

3D View. DOI: 10.1107/S2414314620014078/wm4141sup4.tif

Supporting information file. DOI: 10.1107/S2414314620014078/wm4141Isup5.mol

CCDC reference: 2039861

Additional supporting information:  crystallographic information; 3D view; checkCIF report

## Figures and Tables

**Table 1:** Selected geometric parameters (Å, °)

Cu1—O5^i^	2.376 (2)	Cu1—N1	1.989 (2)
Cu1—O3	2.333 (2)	Cu1—N3	2.014 (2)
Cu1—N2	1.985 (2)	Cu1—N4	2.012 (2)
			
N2—Cu1—O5^i^	90.71 (8)	N1—Cu1—N3	94.85 (10)
N2—Cu1—O3	94.76 (9)	N1—Cu1—N4	176.62 (9)
N2—Cu1—N1	81.56 (9)	N3—Cu1—O5^i^	87.89 (9)
N2—Cu1—N3	175.99 (10)	N3—Cu1—O3	87.27 (9)
N2—Cu1—N4	95.12 (9)	N4—Cu1—O5^i^	83.46 (9)
N1—Cu1—O5^i^	95.88 (8)	N4—Cu1—O3	86.38 (9)
N1—Cu1—O3	94.53 (9)	N4—Cu1—N3	88.45 (10)

Symmetry code: (i) 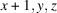 .

**Table 2:** Hydrogen-bond geometry (Å, °)

*D*—H⋯*A*	*D*—H	H⋯*A*	*D*⋯*A*	*D*—H⋯*A*
C7—H7⋯O4^ii^	0.95	2.23	3.175 (4)	177
C4—H4⋯O4^ii^	0.95	2.37	3.315 (3)	175
C9—H9⋯O7	0.95	2.32	3.185 (4)	151
C16—H16*A*⋯O6	0.98	2.38	3.170 (4)	138
C14—H14*A*⋯O8^iii^	0.98	2.35	3.194 (4)	144

Symmetry codes: (ii) 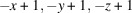 ; (iii) 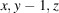 .

**Table 3:** Experimental details

Crystal data	
Chemical formula	[Cu(CF_3_SO_3_)(C_2_H_3_N)_2_(C_12_H_12_N_2_O_2_)](CF_3_O_3_S)
*M*_r_	660.02
Crystal system, space group	Triclinic, *P* 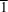
Temperature (K)	100
*a*, *b*, *c* (Å)	7.1004 (2), 12.0708 (4), 14.8155 (4)
α, β, γ (°)	87.368 (2), 89.436 (2), 76.819 (3)
*V* (Å^3^)	1235.04 (6)
*Z*	2
Radiation type	Mo *K*α
μ (mm^−1^)	1.15
Crystal size (mm)	0.40 × 0.10 × 0.07

Data collection	
Diffractometer	XtaLAB AFC12 (RCD3): Kappa single
Absorption correction	Multi-scan (*CrysAlis PRO*; Rigaku OD, 2019 ▸)
*T*_min_, *T*_max_	0.884, 1.000
No. of measured, independent and observed [*I* > 2σ(*I*)] reflections	37314, 5666, 5392
*R*_int_	0.040
(sin θ/λ)_max_ (Å^−1^)	0.650

Refinement	
*R*[*F* ^2^ > 2σ(*F* ^2^)], *wR*(*F* ^2^), *S*	0.049, 0.125, 1.07
No. of reflections	5666
No. of parameters	356
H-atom treatment	H-atom parameters constrained
Δρ_max_, Δρ_min_ (e Å^−3^)	2.01, −0.46

Computer programs: *CrysAlis PRO* (Rigaku OD, 2019 ▸), *SHELXT* (Sheldrick, 2015*a* ▸), *SHELXL* (Sheldrick, 2015*b* ▸) and *OLEX2* (Dolomanov *et al.*, 2009 ▸).

## full crystallographic data

### Crystal data

**Table:**

[Cu(CF_3_SO_3_)(C_2_H_3_N)_2_(C_12_H_12_N_2_O_2_)](CF_3_O_3_S)	*Z* = 2
*M**_r_* = 660.02	*F*(000) = 666
Triclinic, *P*1	*D*_x_ = 1.775 Mg m^−^^3^
*a* = 7.1004 (2) Å	Mo *K*α radiation, λ = 0.71073 Å
*b* = 12.0708 (4) Å	Cell parameters from 13491 reflections
*c* = 14.8155 (4) Å	θ = 2.7–29.0°
α = 87.368 (2)°	µ = 1.15 mm^−^^1^
β = 89.436 (2)°	*T* = 100 K
γ = 76.819 (3)°	Plate, clear light blue
*V* = 1235.04 (6) Å^3^	0.40 × 0.10 × 0.07 mm

### Data collection

**Table:**

XtaLAB AFC12 (RCD3): Kappa single diffractometer	5666 independent reflections
Radiation source: Rotating-anode X-ray tube, Rigaku (Mo) X-ray Source	5392 reflections with *I* > 2σ(*I*)
Mirror monochromator	*R*_int_ = 0.040
ω scans	θ_max_ = 27.5°, θ_min_ = 2.2°
Absorption correction: multi-scan (CrysAlisPro; Rigaku OD, 2019)	*h* = −9→9
*T*_min_ = 0.884, *T*_max_ = 1.000	*k* = −15→15
37314 measured reflections	*l* = −18→19

### Refinement

**Table:**

Refinement on *F*^2^	0 restraints
Least-squares matrix: full	Hydrogen site location: inferred from neighbouring sites
*R*[*F*^2^ > 2σ(*F*^2^)] = 0.049	H-atom parameters constrained
*wR*(*F*^2^) = 0.125	*w* = 1/[σ^2^(*F*_o_^2^) + (0.060*P*)^2^ + 2.9*P*] where *P* = (*F*_o_^2^ + 2*F*_c_^2^)/3
*S* = 1.07	(Δ/σ)_max_ = 0.001
5666 reflections	Δρ_max_ = 2.01 e Å^−^^3^
356 parameters	Δρ_min_ = −0.46 e Å^−^^3^

### Special details

**Table:**

Geometry. All esds (except the esd in the dihedral angle between two l.s. planes) are estimated using the full covariance matrix. The cell esds are taken into account individually in the estimation of esds in distances, angles and torsion angles; correlations between esds in cell parameters are only used when they are defined by crystal symmetry. An approximate (isotropic) treatment of cell esds is used for estimating esds involving l.s. planes.

### Fractional atomic coordinates and isotropic or equivalent isotropic displacement parameters (Å^2^)

**Table:**

	*x*	*y*	*z*	*U*_iso_*/*U*_eq_	
Cu1	0.75757 (5)	0.42109 (3)	0.78071 (2)	0.01474 (11)	
S1	0.23675 (10)	0.48568 (6)	0.76732 (4)	0.01596 (15)	
S2	0.61504 (12)	0.95110 (6)	0.80753 (5)	0.02364 (18)	
F3	0.3180 (3)	0.67475 (15)	0.81898 (12)	0.0238 (4)	
F2	0.0138 (3)	0.67978 (15)	0.81561 (13)	0.0247 (4)	
F1	0.1895 (3)	0.58506 (16)	0.92313 (12)	0.0299 (4)	
O5	0.0885 (3)	0.42653 (17)	0.79453 (14)	0.0197 (4)	
O3	0.4282 (3)	0.42680 (17)	0.79606 (15)	0.0215 (4)	
O4	0.2216 (4)	0.53393 (19)	0.67652 (14)	0.0315 (6)	
O2	0.6860 (3)	0.74737 (17)	0.44968 (14)	0.0244 (5)	
O1	0.8576 (3)	0.12536 (17)	0.45169 (14)	0.0238 (5)	
F5	0.3647 (4)	1.0672 (2)	0.91972 (16)	0.0500 (6)	
N2	0.7231 (3)	0.53835 (19)	0.68011 (15)	0.0143 (4)	
F6	0.4294 (4)	1.16541 (18)	0.80405 (19)	0.0536 (7)	
N1	0.7950 (3)	0.3178 (2)	0.67802 (16)	0.0151 (4)	
O6	0.5595 (4)	0.84920 (19)	0.84105 (17)	0.0369 (6)	
O7	0.6329 (4)	0.9614 (2)	0.71066 (16)	0.0336 (6)	
N3	0.8040 (4)	0.2945 (2)	0.87692 (16)	0.0198 (5)	
F4	0.2473 (4)	1.0479 (2)	0.7904 (2)	0.0594 (7)	
N4	0.7207 (4)	0.5332 (2)	0.87937 (16)	0.0176 (5)	
C6	0.7481 (4)	0.4965 (2)	0.59648 (18)	0.0146 (5)	
C5	0.7856 (4)	0.3705 (2)	0.59509 (18)	0.0147 (5)	
O8	0.7668 (4)	0.9828 (3)	0.8563 (2)	0.0505 (8)	
C7	0.7353 (4)	0.5661 (2)	0.51903 (19)	0.0177 (5)	
H7	0.752622	0.534456	0.461161	0.021*	
C15	0.7130 (4)	0.5956 (2)	0.93529 (18)	0.0175 (6)	
C4	0.8068 (4)	0.3119 (2)	0.51601 (19)	0.0165 (5)	
H4	0.801421	0.351339	0.458761	0.020*	
C17	0.1866 (4)	0.6126 (2)	0.83457 (19)	0.0181 (5)	
C9	0.6697 (4)	0.7277 (2)	0.61315 (19)	0.0172 (5)	
H9	0.641922	0.807505	0.620840	0.021*	
C8	0.6961 (4)	0.6845 (2)	0.52759 (19)	0.0176 (5)	
C13	0.8214 (4)	0.2295 (2)	0.9359 (2)	0.0202 (6)	
C10	0.6850 (4)	0.6514 (2)	0.68725 (19)	0.0161 (5)	
H10	0.667564	0.680938	0.745858	0.019*	
C3	0.8363 (4)	0.1932 (2)	0.52261 (19)	0.0181 (6)	
C2	0.8441 (5)	0.1391 (2)	0.6079 (2)	0.0208 (6)	
H2	0.863236	0.058601	0.614073	0.025*	
C1	0.8236 (4)	0.2034 (2)	0.68297 (19)	0.0184 (6)	
H1	0.829999	0.165612	0.740916	0.022*	
C16	0.7075 (5)	0.6759 (3)	1.0062 (2)	0.0272 (7)	
H16A	0.681353	0.753596	0.979411	0.041*	
H16B	0.832382	0.659186	1.037540	0.041*	
H16C	0.605032	0.668817	1.049419	0.041*	
C14	0.8362 (5)	0.1454 (3)	1.0107 (2)	0.0274 (7)	
H14A	0.822734	0.072737	0.987782	0.041*	
H14B	0.733475	0.171786	1.054573	0.041*	
H14C	0.962531	0.135124	1.040026	0.041*	
C11	0.8548 (5)	0.1775 (3)	0.3622 (2)	0.0233 (6)	
H11A	0.869985	0.118684	0.317564	0.035*	
H11B	0.961202	0.216662	0.356008	0.035*	
H11C	0.731362	0.232593	0.352245	0.035*	
C18	0.4039 (5)	1.0638 (3)	0.8315 (2)	0.0309 (7)	
C12	0.6424 (5)	0.8692 (2)	0.4558 (2)	0.0261 (7)	
H12A	0.633264	0.905278	0.394865	0.039*	
H12B	0.745317	0.891233	0.489377	0.039*	
H12C	0.518965	0.894066	0.487237	0.039*	

### Atomic displacement parameters (Å^2^)

**Table:**

	*U*^11^	*U*^22^	*U*^33^	*U*^12^	*U*^13^	*U*^23^
Cu1	0.02004 (19)	0.01336 (18)	0.01075 (17)	−0.00366 (13)	−0.00062 (12)	−0.00033 (12)
S1	0.0205 (3)	0.0159 (3)	0.0118 (3)	−0.0046 (3)	0.0001 (2)	−0.0019 (2)
S2	0.0348 (4)	0.0170 (3)	0.0196 (4)	−0.0064 (3)	−0.0015 (3)	−0.0030 (3)
F3	0.0251 (9)	0.0198 (9)	0.0292 (10)	−0.0097 (7)	−0.0002 (7)	−0.0047 (7)
F2	0.0228 (9)	0.0184 (8)	0.0310 (10)	−0.0005 (7)	−0.0001 (7)	−0.0025 (7)
F1	0.0532 (13)	0.0235 (9)	0.0120 (8)	−0.0063 (9)	0.0011 (8)	−0.0025 (7)
O5	0.0225 (10)	0.0133 (9)	0.0237 (10)	−0.0048 (8)	−0.0003 (8)	−0.0009 (8)
O3	0.0226 (11)	0.0142 (10)	0.0278 (11)	−0.0039 (8)	0.0009 (9)	−0.0017 (8)
O4	0.0613 (17)	0.0207 (11)	0.0113 (10)	−0.0068 (11)	0.0002 (10)	−0.0001 (8)
O2	0.0413 (13)	0.0137 (10)	0.0174 (10)	−0.0048 (9)	−0.0019 (9)	0.0026 (8)
O1	0.0406 (13)	0.0139 (10)	0.0159 (10)	−0.0035 (9)	−0.0011 (9)	−0.0025 (8)
F5	0.0767 (18)	0.0344 (12)	0.0375 (13)	−0.0086 (12)	0.0220 (12)	−0.0112 (10)
N2	0.0167 (11)	0.0141 (11)	0.0123 (10)	−0.0037 (9)	−0.0011 (8)	−0.0015 (8)
F6	0.0781 (18)	0.0147 (10)	0.0640 (17)	−0.0041 (11)	0.0229 (14)	0.0023 (10)
N1	0.0169 (11)	0.0144 (11)	0.0141 (11)	−0.0037 (9)	−0.0006 (9)	−0.0008 (8)
O6	0.0630 (18)	0.0161 (11)	0.0296 (13)	−0.0063 (11)	0.0090 (12)	0.0034 (9)
O7	0.0578 (17)	0.0232 (12)	0.0214 (12)	−0.0130 (11)	0.0093 (11)	−0.0020 (9)
N3	0.0271 (13)	0.0184 (12)	0.0141 (11)	−0.0054 (10)	−0.0006 (10)	−0.0004 (9)
F4	0.0390 (14)	0.0580 (17)	0.077 (2)	0.0013 (12)	−0.0125 (13)	−0.0180 (14)
N4	0.0198 (12)	0.0176 (11)	0.0149 (11)	−0.0032 (9)	0.0004 (9)	0.0007 (9)
C6	0.0155 (12)	0.0150 (13)	0.0133 (12)	−0.0034 (10)	0.0004 (10)	−0.0015 (10)
C5	0.0144 (12)	0.0153 (13)	0.0146 (12)	−0.0039 (10)	−0.0011 (10)	0.0000 (10)
O8	0.0450 (17)	0.0538 (18)	0.0533 (18)	−0.0083 (14)	−0.0141 (14)	−0.0214 (15)
C7	0.0246 (14)	0.0161 (13)	0.0126 (12)	−0.0042 (11)	−0.0014 (10)	−0.0021 (10)
C15	0.0216 (14)	0.0168 (13)	0.0124 (12)	−0.0017 (11)	0.0003 (10)	0.0035 (10)
C4	0.0197 (13)	0.0137 (12)	0.0152 (13)	−0.0023 (10)	−0.0007 (10)	0.0004 (10)
C17	0.0218 (14)	0.0185 (13)	0.0145 (13)	−0.0050 (11)	0.0014 (10)	−0.0022 (10)
C9	0.0197 (13)	0.0122 (12)	0.0195 (14)	−0.0033 (10)	−0.0015 (11)	−0.0009 (10)
C8	0.0197 (13)	0.0167 (13)	0.0167 (13)	−0.0048 (11)	−0.0012 (10)	0.0010 (10)
C13	0.0248 (15)	0.0170 (13)	0.0189 (14)	−0.0042 (11)	−0.0025 (11)	−0.0036 (11)
C10	0.0173 (13)	0.0158 (13)	0.0152 (13)	−0.0035 (10)	0.0007 (10)	−0.0028 (10)
C3	0.0209 (14)	0.0157 (13)	0.0171 (13)	−0.0028 (11)	−0.0007 (11)	−0.0031 (10)
C2	0.0287 (15)	0.0118 (12)	0.0211 (14)	−0.0033 (11)	0.0001 (12)	−0.0003 (10)
C1	0.0241 (14)	0.0146 (13)	0.0156 (13)	−0.0033 (11)	−0.0006 (11)	0.0031 (10)
C16	0.0411 (19)	0.0219 (15)	0.0171 (14)	−0.0038 (13)	−0.0012 (13)	−0.0038 (12)
C14	0.045 (2)	0.0185 (14)	0.0191 (15)	−0.0086 (13)	−0.0028 (13)	0.0019 (11)
C11	0.0338 (17)	0.0190 (14)	0.0151 (14)	−0.0016 (12)	0.0001 (12)	−0.0023 (11)
C18	0.0414 (19)	0.0191 (15)	0.0317 (18)	−0.0058 (14)	0.0046 (15)	−0.0012 (13)
C12	0.0390 (18)	0.0121 (13)	0.0260 (16)	−0.0042 (12)	−0.0032 (13)	0.0031 (11)

### Geometric parameters (Å, º)

**Table:**

Cu1—O5^i^	2.376 (2)	C6—C5	1.484 (4)
Cu1—O3	2.333 (2)	C6—C7	1.382 (4)
Cu1—N2	1.985 (2)	C5—C4	1.386 (4)
Cu1—N1	1.989 (2)	C7—H7	0.9500
Cu1—N3	2.014 (2)	C7—C8	1.403 (4)
Cu1—N4	2.012 (2)	C15—C16	1.456 (4)
S1—O5	1.445 (2)	C4—H4	0.9500
S1—O3	1.442 (2)	C4—C3	1.399 (4)
S1—O4	1.436 (2)	C9—H9	0.9500
S1—C17	1.831 (3)	C9—C8	1.388 (4)
S2—O6	1.441 (2)	C9—C10	1.389 (4)
S2—O7	1.442 (2)	C13—C14	1.456 (4)
S2—O8	1.435 (3)	C10—H10	0.9500
S2—C18	1.824 (4)	C3—C2	1.391 (4)
F3—C17	1.336 (3)	C2—H2	0.9500
F2—C17	1.332 (3)	C2—C1	1.374 (4)
F1—C17	1.338 (3)	C1—H1	0.9500
O2—C8	1.345 (3)	C16—H16A	0.9800
O2—C12	1.439 (3)	C16—H16B	0.9800
O1—C3	1.348 (3)	C16—H16C	0.9800
O1—C11	1.439 (3)	C14—H14A	0.9800
F5—C18	1.335 (4)	C14—H14B	0.9800
N2—C6	1.354 (3)	C14—H14C	0.9800
N2—C10	1.339 (4)	C11—H11A	0.9800
F6—C18	1.325 (4)	C11—H11B	0.9800
N1—C5	1.353 (3)	C11—H11C	0.9800
N1—C1	1.348 (4)	C12—H12A	0.9800
N3—C13	1.136 (4)	C12—H12B	0.9800
F4—C18	1.328 (5)	C12—H12C	0.9800
N4—C15	1.138 (4)		
			
O3—Cu1—O5^i^	168.85 (8)	F2—C17—S1	112.0 (2)
N2—Cu1—O5^i^	90.71 (8)	F2—C17—F3	107.2 (2)
N2—Cu1—O3	94.76 (9)	F2—C17—F1	107.3 (2)
N2—Cu1—N1	81.56 (9)	F1—C17—S1	111.5 (2)
N2—Cu1—N3	175.99 (10)	C8—C9—H9	120.8
N2—Cu1—N4	95.12 (9)	C8—C9—C10	118.3 (3)
N1—Cu1—O5^i^	95.88 (8)	C10—C9—H9	120.8
N1—Cu1—O3	94.53 (9)	O2—C8—C7	115.7 (3)
N1—Cu1—N3	94.85 (10)	O2—C8—C9	125.2 (3)
N1—Cu1—N4	176.62 (9)	C9—C8—C7	119.1 (3)
N3—Cu1—O5^i^	87.89 (9)	N3—C13—C14	177.8 (3)
N3—Cu1—O3	87.27 (9)	N2—C10—C9	123.2 (3)
N4—Cu1—O5^i^	83.46 (9)	N2—C10—H10	118.4
N4—Cu1—O3	86.38 (9)	C9—C10—H10	118.4
N4—Cu1—N3	88.45 (10)	O1—C3—C4	124.8 (3)
O5—S1—C17	104.08 (13)	O1—C3—C2	116.3 (3)
O3—S1—O5	113.46 (12)	C2—C3—C4	118.8 (3)
O3—S1—C17	103.30 (13)	C3—C2—H2	120.4
O4—S1—O5	115.66 (15)	C1—C2—C3	119.2 (3)
O4—S1—O3	115.70 (15)	C1—C2—H2	120.4
O4—S1—C17	102.27 (13)	N1—C1—C2	122.8 (3)
O6—S2—O7	114.84 (15)	N1—C1—H1	118.6
O6—S2—C18	103.34 (16)	C2—C1—H1	118.6
O7—S2—C18	103.15 (16)	C15—C16—H16A	109.5
O8—S2—O6	115.89 (19)	C15—C16—H16B	109.5
O8—S2—O7	114.17 (19)	C15—C16—H16C	109.5
O8—S2—C18	103.02 (18)	H16A—C16—H16B	109.5
S1—O5—Cu1^ii^	145.62 (13)	H16A—C16—H16C	109.5
S1—O3—Cu1	144.69 (13)	H16B—C16—H16C	109.5
C8—O2—C12	117.2 (2)	C13—C14—H14A	109.5
C3—O1—C11	118.2 (2)	C13—C14—H14B	109.5
C6—N2—Cu1	114.82 (18)	C13—C14—H14C	109.5
C10—N2—Cu1	126.88 (19)	H14A—C14—H14B	109.5
C10—N2—C6	118.3 (2)	H14A—C14—H14C	109.5
C5—N1—Cu1	115.06 (18)	H14B—C14—H14C	109.5
C1—N1—Cu1	126.97 (19)	O1—C11—H11A	109.5
C1—N1—C5	117.9 (2)	O1—C11—H11B	109.5
C13—N3—Cu1	174.2 (2)	O1—C11—H11C	109.5
C15—N4—Cu1	175.4 (2)	H11A—C11—H11B	109.5
N2—C6—C5	114.5 (2)	H11A—C11—H11C	109.5
N2—C6—C7	122.4 (2)	H11B—C11—H11C	109.5
C7—C6—C5	123.1 (2)	F5—C18—S2	111.6 (2)
N1—C5—C6	114.0 (2)	F6—C18—S2	111.7 (3)
N1—C5—C4	122.8 (2)	F6—C18—F5	107.5 (3)
C4—C5—C6	123.2 (2)	F6—C18—F4	107.8 (3)
C6—C7—H7	120.7	F4—C18—S2	111.2 (2)
C6—C7—C8	118.6 (3)	F4—C18—F5	106.9 (3)
C8—C7—H7	120.7	O2—C12—H12A	109.5
N4—C15—C16	178.7 (3)	O2—C12—H12B	109.5
C5—C4—H4	120.8	O2—C12—H12C	109.5
C5—C4—C3	118.3 (3)	H12A—C12—H12B	109.5
C3—C4—H4	120.8	H12A—C12—H12C	109.5
F3—C17—S1	111.09 (19)	H12B—C12—H12C	109.5
F3—C17—F1	107.6 (2)		
			
Cu1—N2—C6—C5	3.3 (3)	O7—S2—C18—F4	60.7 (3)
Cu1—N2—C6—C7	−178.0 (2)	C6—N2—C10—C9	0.0 (4)
Cu1—N2—C10—C9	177.6 (2)	C6—C5—C4—C3	−178.2 (3)
Cu1—N1—C5—C6	−0.2 (3)	C6—C7—C8—O2	179.9 (3)
Cu1—N1—C5—C4	−179.5 (2)	C6—C7—C8—C9	−0.6 (4)
Cu1—N1—C1—C2	178.6 (2)	C5—N1—C1—C2	0.2 (4)
O5—S1—O3—Cu1	169.10 (19)	C5—C6—C7—C8	179.0 (3)
O5—S1—C17—F3	177.75 (19)	C5—C4—C3—O1	179.4 (3)
O5—S1—C17—F2	−62.4 (2)	C5—C4—C3—C2	−0.3 (4)
O5—S1—C17—F1	57.8 (2)	O8—S2—C18—F5	−61.0 (3)
O3—S1—O5—Cu1^ii^	−171.80 (19)	O8—S2—C18—F6	59.4 (3)
O3—S1—C17—F3	59.0 (2)	O8—S2—C18—F4	179.7 (3)
O3—S1—C17—F2	178.83 (19)	C7—C6—C5—N1	179.3 (3)
O3—S1—C17—F1	−61.0 (2)	C7—C6—C5—C4	−1.5 (4)
O4—S1—O5—Cu1^ii^	−34.7 (3)	C4—C3—C2—C1	−0.4 (4)
O4—S1—O3—Cu1	32.0 (3)	C17—S1—O5—Cu1^ii^	76.6 (2)
O4—S1—C17—F3	−61.5 (2)	C17—S1—O3—Cu1	−78.9 (2)
O4—S1—C17—F2	58.3 (2)	C8—C9—C10—N2	−0.3 (4)
O4—S1—C17—F1	178.5 (2)	C10—N2—C6—C5	−178.8 (2)
O1—C3—C2—C1	179.9 (3)	C10—N2—C6—C7	−0.1 (4)
N2—C6—C5—N1	−2.0 (3)	C10—C9—C8—O2	−180.0 (3)
N2—C6—C5—C4	177.2 (3)	C10—C9—C8—C7	0.6 (4)
N2—C6—C7—C8	0.4 (4)	C3—C2—C1—N1	0.4 (5)
N1—C5—C4—C3	1.0 (4)	C1—N1—C5—C6	178.3 (2)
O6—S2—C18—F5	60.1 (3)	C1—N1—C5—C4	−0.9 (4)
O6—S2—C18—F6	−179.6 (3)	C11—O1—C3—C4	1.6 (4)
O6—S2—C18—F4	−59.2 (3)	C11—O1—C3—C2	−178.7 (3)
O7—S2—C18—F5	180.0 (3)	C12—O2—C8—C7	178.8 (3)
O7—S2—C18—F6	−59.7 (3)	C12—O2—C8—C9	−0.6 (4)

Symmetry codes: (i) *x*+1, *y*, *z*; (ii) *x*−1, *y*, *z*.

### Hydrogen-bond geometry (Å, º)

**Table:**

*D*—H···*A*	*D*—H	H···*A*	*D*···*A*	*D*—H···*A*
C7—H7···O4^iii^	0.95	2.23	3.175 (4)	177
C4—H4···O4^iii^	0.95	2.37	3.315 (3)	175
C9—H9···O7	0.95	2.32	3.185 (4)	151
C16—H16*A*···O6	0.98	2.38	3.170 (4)	138
C14—H14*A*···O8^iv^	0.98	2.35	3.194 (4)	144

Symmetry codes: (iii) −*x*+1, −*y*+1, −*z*+1; (iv) *x*, *y*−1, *z*.
