# Supplementary figures and images for: catena-Poly[[[bis­(aceto­nitrile-κN)(4,4′-dimeth­oxy-2,2′-bi­pyridine-κ2 N,N′)copper(II)]-μ-tri­fluoro­methane­sulfonato-κ2 O:O′] tri­fluoro­methane­sulfonate]
Source: IUCrdata. 2020 Oct 30;5(Pt 10):x201407. doi: 10.1107/S2414314620014078 (PMC9462164; doi:10.1107/S2414314620014078)

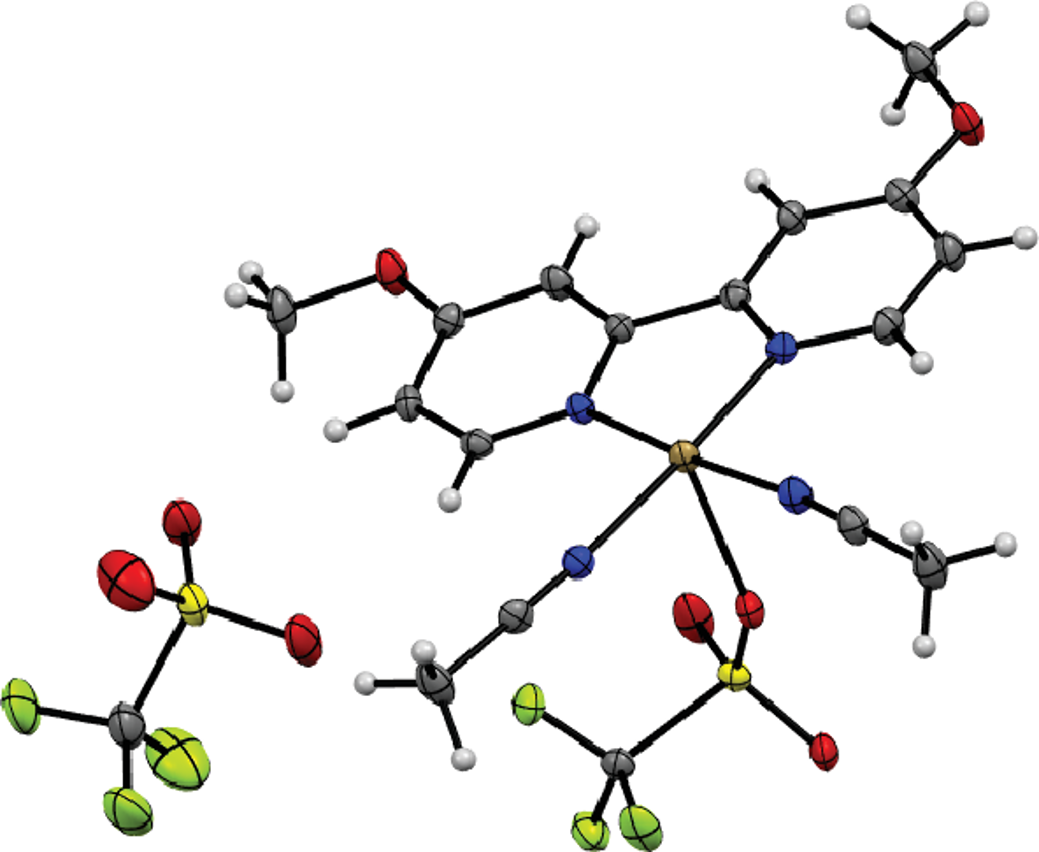

Supplement: Supplementary file 4 [file x-05-x201407-sup4.tif]
